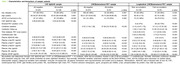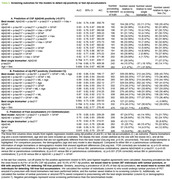# Plasma biomarkers combinations for prescreening rapid amyloid accumulation in cognitively unimpaired individuals at‐risk of Alzheimer’s disease

**DOI:** 10.1002/alz.091423

**Published:** 2025-01-09

**Authors:** José Contador, Marta Milà‐Alomà, Armand González Escalante, Nicholas J. Ashton, Mahnaz Shekari, Paula Ortiz‐Romero, Thomas K Karikari, Eugeen Vanmechelen, Theresa A. Day, Jeffrey L. Dage, Henrik Zetterberg, Juan Domingo Gispert, Kaj Blennow, Marc Suarez‐Calvet

**Affiliations:** ^1^ Barcelonaβeta Brain Research Center (BBRC), Pasqual Maragall Foundation, Barcelona Spain; ^2^ Cognitive Decline and Movement Disorders Unit, Neurology Department, Hospital del Mar, Barcelona Spain; ^3^ Hospital del Mar Research Institute, Barcelona, Barcelona Spain; ^4^ Universitat Pompeu Fabra, Barcelona Spain; ^5^ Centro de Investigación Biomédica en Red de Fragilidad y Envejecimiento Saludable (CIBERFES), Madrid Spain; ^6^ Hospital del Mar Research Institute (IMIM), Barcelona Spain; ^7^ NIHR Biomedical Research Centre for Mental Health & Biomedical Research Unit for Dementia at South London & Maudsley NHS Foundation, London United Kingdom; ^8^ Wallenberg Centre for Molecular and Translational Medicine, University of Gothenburg, Gothenburg Sweden; ^9^ King’s College London, Institute of Psychiatry, Psychology & Neuroscience, Maurice Wohl Clinical Neuroscience Institute, London United Kingdom; ^10^ Department of Psychiatry and Neurochemistry, Institute of Neuroscience and Physiology, The Sahlgrenska Academy, University of Gothenburg, Mölndal, Gothenburg Sweden; ^11^ University of Pittsburgh, Pittsburgh, PA USA; ^12^ ADx NeuroSciences NV, Ghent Belgium; ^13^ Lilly Research Laboratories, Eli Lilly and Company, Indianapolis, IN USA; ^14^ Stark Neurosciences Research Institute, Indiana University School of Medicine, Indiana, IN USA; ^15^ Department of Neurodegenerative Disease, UCL Queen Square Institute of Neurology, University College London, London, ‐ United Kingdom; ^16^ Clinical Neurochemistry Laboratory, Sahlgrenska University Hospital, Mölndal Sweden; ^17^ Department of Psychiatry and Neurochemistry, Institute of Neuroscience and Physiology, The Sahlgrenska Academy at the University of Gothenburg, Mölndal Sweden; ^18^ UK Dementia Research Institute at UCL, London United Kingdom; ^19^ Centro de Investigación Biomédica en Red Bioingeniería, Biomateriales y Nanomedicina (CIBER‐BBN), Instituto de Salud Carlos III, Madrid Spain

## Abstract

**Background:**

Alzheimer’s disease (AD) blood biomarkers alone can detect amyloid‐β (Aβ) pathology in cognitively unimpaired (CU) individuals. We assessed whether combining different plasma biomarkers improves the detection of Aβ‐positivity and identifies rapid amyloid deposition in CU individuals.

**Method:**

CU participants from the ALFA+ cohort were included. Among them, 361 had CSF Aβ42/40 and 328 amyloid PET‐scans [194 with two longitudinal scans; mean interval=3.35 (0.56) years]. Plasma Aβ42/40, p‐tau181, p‐tau231, GFAP, NfL (Simoa‐based) and p‐tau217 and t‐tau (MSD‐based) were measured at baseline (Table 1). We used simple and multiple logistic models to estimate Aβ‐positivity (defined as CSF Aβ42/40<0.071 or amyloid‐PET>12 Centiloids) or Aβ accumulation rate (“Fast accumulators” defined as >3 Centiloids/year). The model contained plasma biomarkers and demographics (age and sex) as covariates. We selected as "best model" (BM) that with lowest AIC. We defined parsimonious models as those with an AUC not significantly different (DeLong test) from BM or from each other yet outperforming single biomarkers and/or demographics models (FDR corrected). For the positive agreement closest to 90%, we calculated savings in lumbar punctures and amyloid PET‐scans.

**Result:**

For CSF Aβ‐positive detection, BM included plasma Aβ42/40, p‐tau181, p‐tau217, p‐tau231, GFAP and t‐tau (AUC=0.84). All simpler biomarkers combinations included plasma Ab42/40 and p‐tau231 (Table 2A). For PET Ab‐positive detection, BM included plasma Aβ42/40, p‐tau181, p‐tau217, GFAP, NFL and age (AUC=0.88). All simpler biomarkers combinations included plasma Ab42/40 and p‐tau217 (Table 2B). Regarding fast accumulators’ detection, plasma p‐tau217 was the single biomarker with the highest performance (AUC=0.70). BM included plasma Aβ42/40, p‐tau217, p‐tau231 and GFAP (AUC= 0.76). BM and the plasma Aβ42/40, p‐tau217 and GFAP (AUC=0.75) combination were the only models that outperformed the age and sex combination and single biomarkers, except for plasma p‐tau217, Aβ42/40 (AUC=0.69) or GFAP (AUC=0.68) alone (Table 2C). The combination of biomarkers could save up to 11% of lumbar punctures or 44% of amyloid‐PET to detect Ab‐positive CU individuals and 16% amyloid‐PETs to detect fast Aβ‐accumulation compared to the best single plasma biomarker (Table 2).

**Conclusion:**

In CU individuals, diverse combinations of plasma biomarkers detect Aβ‐positivity and future Aβ‐accumulation with high accuracy and can lead to substantial cost savings in AD detection.